# Clinical Diagnosis, Treatment, and Outcome Analysis of a Horse with Proximal Sesamoid Bone Fracture Complicated by Flexor Tendinitis

**DOI:** 10.3390/vetsci13010040

**Published:** 2026-01-02

**Authors:** Zhiyuan Zhang, Yang Yang, Yuhui Ma, Zhanhai Mai, Han Fu, Xutian Wang, Xiongjian Cao, Tianqing Li, Jianlong Li, Qingyong Guo

**Affiliations:** 1 Xinjiang Key Laboratory of New Drug Research and Development for Herbivorous (XJ-LNDRDH), College of Animal Medicine, Xinjiang Agricultural University, Urumqi 830052, China; 15299943180@163.com (Z.Z.); sdwfyy9809@163.com (Y.Y.); mzh881231@126.com (Z.M.); fh971108@163.com (H.F.); xvt9981@163.com (X.W.); psc9857@163.com (X.C.); 13199816414@163.com (T.L.); 2 Xingjiang Zhaosu County Xiyu Horse Industry Co., Ltd., Yili 835600, China; mayuhuim@126.com

**Keywords:** horse, proximal sesamoid bone fracture, flexor tendinitis, low-intensity pulsed ultrasound (LIPUS), diagnostic imaging

## Abstract

With the growing popularity of equestrian sports, the incidence of athletic injuries in horses has also risen. Among these injuries, proximal sesamoid bone fracture (PSBF) and flexor tendinitis are particularly common in the forelimbs of sport horses and represent major causes of musculoskeletal impairment. A 5-year-old horse presented with obvious symptoms such as swelling at the left fetlock joint and metacarpal region after exercise. Through lameness assessment, diagnostic imaging, and hematological testing, the horse was diagnosed with PSBF complicated by flexor tendinitis. The affected horse was treated with non-steroidal anti-inflammatory drugs combined with low-intensity pulsed ultrasound (LIPUS) therapy. After treatment, local microcirculation at the fracture and flexor tendon sites was improved, tissue healing was accelerated, and clinical indicators were stabilized. This case report demonstrates the potential of LIPUS-assisted therapy in promoting the recovery of horses with PSBF and concurrent flexor tendinitis, providing a valuable clinical reference for the management of complex musculoskeletal injuries in veterinary practice.

## Simple Summary

With the increasing popularity of equestrian sports, proximal sesamoid bone fracture (PSBF) and flexor tendinitis in the forelimbs of sport horses have become increasingly common, often leading to musculoskeletal injuries. A 5-year-old horse developed swelling in the left fetlock joint and metacarpal region after exercise and was diagnosed with concurrent PSBF and flexor tendinitis. To promote recovery, the veterinarian administered non-steroidal anti-inflammatory drugs (NSAIDs) in combination with low-intensity pulsed ultrasound (LIPUS) therapy. This treatment improved local microcirculation at the affected sites, accelerated tissue healing, and led to stable recovery of clinical parameters. This case highlights the effectiveness of LIPUS-assisted therapy in managing complex injuries of this kind, providing practical guidance for veterinarians and contributing to the health and sustainable development of equestrian sports.

## 1. Introduction

Fracture of the proximal sesamoid bone (PSB) is a serious and potentially life-threatening musculoskeletal injury in racehorses [1]. Proximal sesamoid bone fracture (PSBF) most frequently occurs in the forelimbs of racehorses and represents a major cause of fatal outcomes associated with musculoskeletal injuries [2,3,4]. The PSB is connected to the distal palmar and plantar surfaces of the third metacarpal (MC3) and metatarsal bones, serving as a fulcrum in the suspensory apparatus. Fracture of the PSB disrupts this support mechanism, leading to failure of the suspensory apparatus and, consequently, severe lameness or even euthanasia in affected racehorses [5,6,7,8,9].

Tendon injury is another common cause of lameness in racehorses. In particular, flexor tendinitis typically results in chronic lameness and requires prolonged healing and rehabilitation, causing significant economic losses in equestrian sports [10,11,12]. The treatment of equine flexor tendinitis is labor-intensive due to the slow regenerative capacity of tendon tissue. After injury, the composition, structure, and function of tendon fibers cannot be fully restored, which increases the risk of recurrence and limits the horse’s athletic performance [13,14,15].

Low-intensity pulsed ultrasound (LIPUS) is a non-invasive therapeutic ultrasound modality that plays a crucial role in promoting fracture healing, enhancing wound repair, modulating immune regulation, and reducing inflammation [16]. As a form of physical therapy, LIPUS can stimulate the proliferation and differentiation of osteoblasts, and has a significant effect on promoting new bone formation and accelerating fracture repair [17]. In orthopedics and rehabilitation medicine, LIPUS is known to promote bone and soft tissue repair with significant therapeutic effects [18,19].

Currently, the main treatment regimens for PSBF include osteoclast inhibitors, e.g., tiludronate sodium, intra-articular injections, such as triamcinolone acetonide or platelet-rich plasma (PRP), into the metacarpophalangeal joint (MTPJ), orthopedic plate fixation, and radial pressure wave therapy [20]. Small bone fragments in PSBF cases may be removed surgically, and in some equine hospitals abroad, arthroscopic minimally invasive surgery is performed, but such procedures may damage the ligaments surrounding the sesamoid bones [21]. Cryotherapy has also been explored as a potential treatment for flexor tendinitis; however, standardized and evidence-based application protocols remain to be confirmed [22].

This paper reports a clinical case of a horse diagnosed with PSBF complicated by flexor tendinitis, providing details of its basic information, medical history, diagnostic methods, and treatment protocol. It aims to enhance the understanding of PSBF by equine veterinarians and to provide a reference for clinical diagnosis and therapy in equine practice. To our knowledge, this study is the first clinical report on the use of NSAIDs combined with LIPUS for treating flexor tendinitis secondary to PSBF in horses, and it highlights the key advantages of this combined approach, mechanistic complementarity, synergistic therapeutic effects, and practical safety, providing a valuable reference for future clinical applications.

## 2. Basic Information

A 5-year-old male Yili horse sustained an injury to its left forelimb due to continuous high-intensity exercise, resulting in obvious lameness. No immediate treatment was administered after the injury. One month later, the horse was referred to Tianma Cultural Park in Zhaosu County, Yili Kazakh Autonomous Prefecture, Xinjiang Uygur Autonomous Region, China (43°09′ N–43°15′ N) for clinical diagnosis and treatment.

## 3. Diagnosis

### 3.1. Clinical Examination

**Figure 1:**
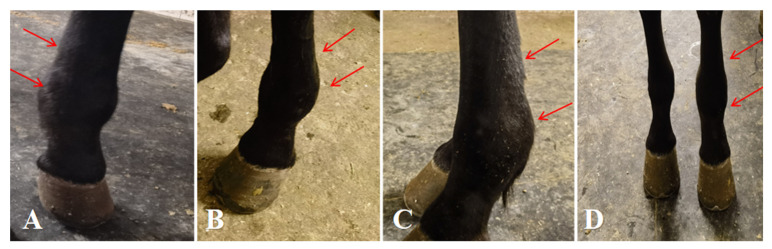
Condition of the affected horse. (**A**–**C**) Proximal sesamoid bone fracture with marked swelling of the fetlock joint and flexor tendons. (**D**) Comparison between the affected and contralateral fetlock joints and flexor tendons. The arrows indicate the specific lesions.

The affected horse was 5 years old and weighed 397 kg, with a body temperature of 38.9 °C and a heart rate of 46 beats per minute. The circumference of the left fetlock joint was 28 cm, compared with 24 cm on the right; while the left cannon bone measured 25 cm and the right 22 cm. Lameness was graded based on previously described criteria [23] (Table 1), with the horse scoring Grade VI. When standing, the horse frequently stamped the ground with its left forelimb. While leading and walking, severe lameness was observed in the left forelimb, accompanied by marked swelling of the fetlock joint and metacarpal region. A distinct pain response was noted when the affected area was palpated (Figure 1).

### 3.2. Imaging Examination

#### 3.2.1. X-Ray Examination

**Figure 2:**
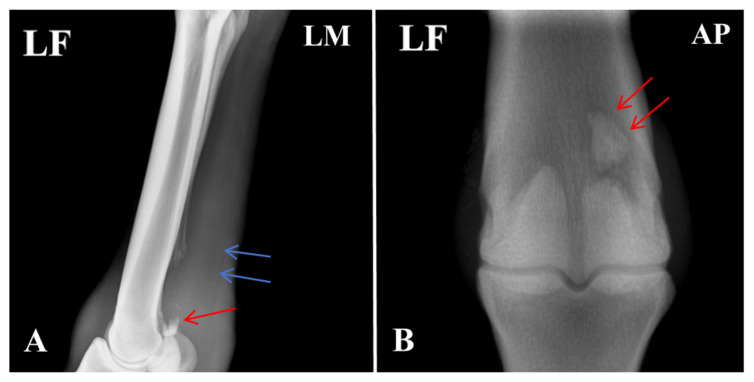
Radiographic features of the affected horse with PSBF. (**A**) Fracture at the apex of the proximal sesamoid bone (red arrow: bone fragments; blue arrow: increased radiodensity in the flexor tendon region). (**B**) Fracture fragments of the proximal sesamoid bone. The arrows indicate the specific lesions.

Radiographic evaluation was performed to assess the type of lesion and to determine whether flexor tendinitis was present. Images were taken of the left forelimb (LF) in lateromedial (LM) and dorsopalmar/anteroposterior (AP) projections. The radiographs showed a clear fracture line at the proximal apex of the left proximal sesamoid bone, with separation of the fracture margins and the presence of a small free bone fragment. The fragment had an irregular shape and was displaced away from the main sesamoid body. Local cortical continuity was disrupted, and the trabecular pattern appeared disorganized. An increase in soft-tissue density was also noted in the flexor tendon region, raising suspicion of concurrent flexor tendinitis (Figure 2).

#### 3.2.2. Ultrasound Examination

**Figure 3:**
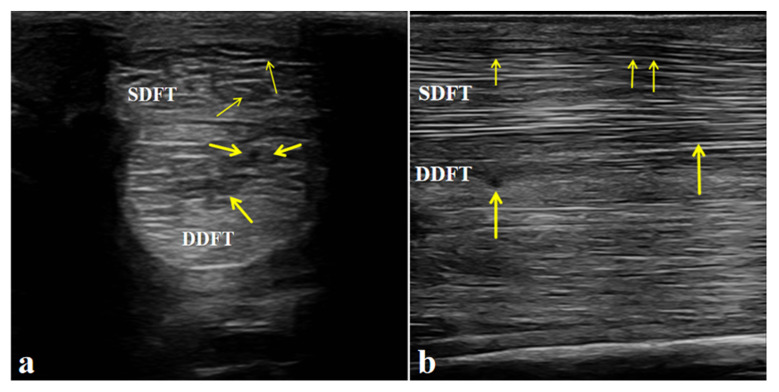
Ultrasonographic features of the affected horse with flexor tendinitis. (**a**) Transverse sonograms of the superficial digital flexor tendon (SDFT) and deep digital flexor tendon (DDFT) (yellow arrow: lesion sites). (**b**) Longitudinal sonograms of the SDFT and DDFT (yellow arrow: lesion sites). Arrows indicate the sites of tendon injury.

Ultrasonographic examination of the affected limb (left forelimb) showed a marked increase in the cross-sectional area of both the superficial digital flexor tendon (SDFT) and the deep digital flexor tendon (DDFT) compared with the contralateral side. The tendon margins were still visible, although slightly roughened. The normal parallel fibrillar echogenic pattern was almost completely lost and replaced by a diffuse, heterogeneous, mildly hypoechoic appearance. These findings indicate mild injury to both the SDFT and DDFT and are consistent with the diagnosis of flexor tendinitis (Figure 3).

### 3.3. Blood Physiological Examination

Blood physiological analysis was performed on the affected horse using automated analyzer BC-5300VET (Mindray Animal Medical Technology Co., Ltd., Shenzhen, China). The results showed elevated levels of inflammatory indicators, including neutrophils (NEU), white blood cells (WBC), and lymphocytes (LYM), indicating a systemic inflammatory response. In contrast, red blood cells (RBC) count, hemoglobin (HGB), mean platelet volume (MPV), and hematocrit (HCT) were decreased, suggesting potential bone marrow suppression, anemia, or dehydration (Table 2).

### 3.4. Blood Biochemical Examination

Blood biochemical analysis was performed on the affected horse using automated analyzer BS-240VET (Mindray Animal Medical Technology Co., Ltd., Shenzhen, China). The results showed elevated levels of creatine kinase (CK) and lactate dehydrogenase (LDH), indicating muscle and soft-tissue injury accompanied by inflammation (Table 3).

### 3.5. Serum Inflammatory Markers Measured by ELISA

Serum inflammatory cytokines were measured using equine-specific ELISA kits for TNF-α, IL-1β, and IL-6 (Yancheng Maiji Biomedical Testing Service Center, Yancheng, China). The results showed elevated levels of TNF-α, IL-1β, and IL-6, suggesting a strong inflammatory response likely associated with the PSBF and concurrent injuries to the SDFT and DDFT (Table 4).

### 3.6. Diagnostic Results

In summary, based on the horse’s clinical signs, medical history, imaging findings, and laboratory test results (hematology, biochemistry, and serum cytokine assays), the horse was diagnosed with PSBF complicated by inflammation of SDFT and DDFT and in the soft tissue surrounding the fractured bone.

## 4. Treatment and Outcome

### 4.1. Treatment Plan

Non-steroidal anti-inflammatory drug (NSAID) therapy combined with low-intensity pulsed ultrasound (LIPUS) adjuvant therapy was adopted for the affected horse. The detailed treatment protocol was as follows.

Treatment started with cold therapy. The affected limb was cooled with either an ice pack or cold-water soaking once daily for 30 min to help reduce acute swelling and inflammation and to prepare the tissues for the following interventions (days 1–7).

Local intra-articular medication was then administered to the fetlock joint. After flexing the limb and disinfecting the area with 75% alcohol, the joint was accessed through the lateral sesamoid–third metacarpal space. Procaine penicillin (6.4 million IU) was injected into the joint cavity to provide local antibacterial and analgesic effects. Through the same entry point, sodium hyaluronate (4 mL per injection) was given every other day for a total of five treatments to improve joint lubrication and protect the articular cartilage (days 1–10).

Precise medications were given according to the horse’s body weight. Flunixin meglumine (1.1 mg/kg) was administered intramuscularly once a day for pain control. Ampicillin sodium (20 mg/kg), dissolved in 4000 mL of 0.9% saline, was given once daily by intravenous infusion to provide systemic anti-inflammatory support, fluid supplementation, and infection prevention. These medications were continued until the acute inflammatory signs had clearly subsided (days 1–7).

LIPUS treatment started at the same time as the medications using HORSELIPUSPRO (transducer model OFS-20257, Beijing Zhouquan Biotechnology Co., Ltd., Beijing, China;) was used. A coupling gel was applied to ensure good contact between the probe and the skin. Using the ED-1001 mode, ultrasound was applied over the fetlock and the injured flexor tendon. The settings were: frequency 1.5 MHz, intensity 50–100 mW/cm^2^, once daily for 15 min. Treatments were carried out for 5 consecutive weeks, completing 35 sessions in total. The aim was to improve the local joint and tendon environment and support tissue repair (days 1–35).

### 4.2. Therapeutic Effect

#### 4.2.1. Post-Treatment Clinical Examination

**Figure 4:**
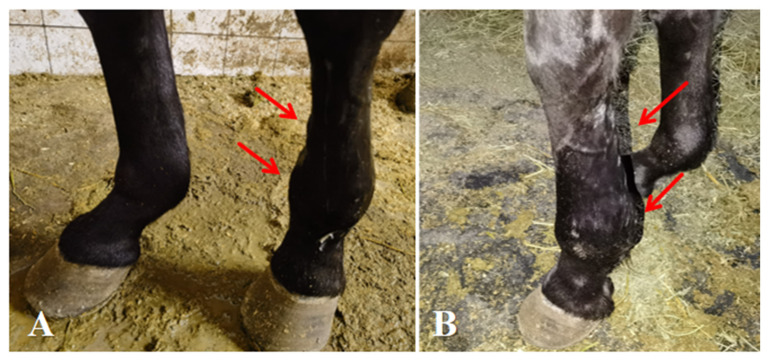
Post-treatment conditions of affected horse. (**A**,**B**) Complete healing of medial and lateral proximal sesamoid bone fractures, with resolution of swelling in the metacarpophalangeal joint and flexor tendons. The arrows indicate the specific lesions.

After five treatment courses over 35 days, the affected horse exhibited the following average indicators: body temperature 37.9 °C, heart rate 36 beats per minute, metacarpophalangeal joint circumference 25 cm (left forelimb) vs. 24 cm (right forelimb), and cannon bone circumference 23 cm (left forelimb) vs. 22 cm (right forelimb). Clinically, the horse maintained a normal state and exhibited no lameness when standing (Figure 4). Lameness during movement was significantly improved. Swelling of the metacarpophalangeal joint had completely subsided, and no tenderness was observed upon palpation. Swelling of the flexor tendons was alleviated, and the lameness grade was improved to Grade II.

#### 4.2.2. Post-Treatment Radiographic Examination

**Figure 5:**
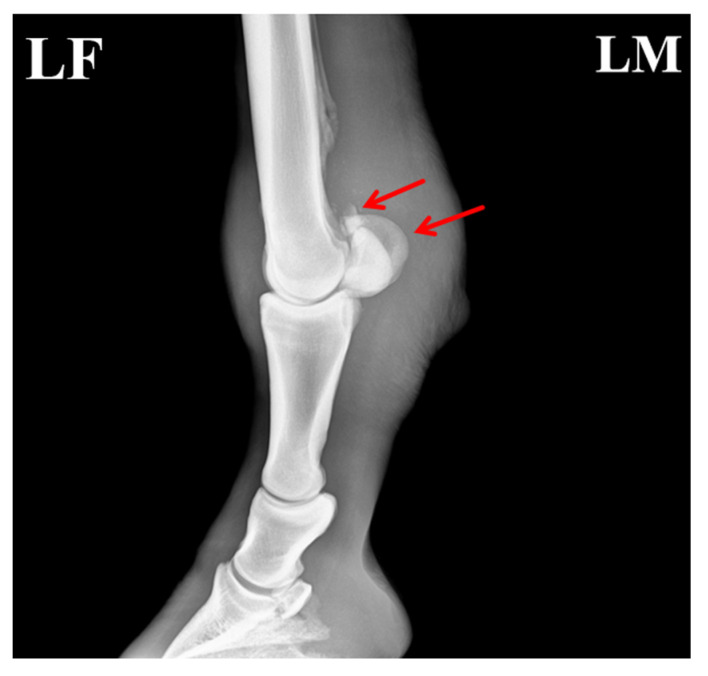
Post-treatment radiograph of the affected horse showing formation of callus at the fracture site. The arrows indicate the specific lesions.

Due to the unique structure and function of the equine sesamoid bones, their healing process is typically more prolonged than that of other fractures. As shown in Figure 5, the proximal apex of the sesamoid bone exhibited a fracture with indistinct and blurred fracture margins, along with a localized area of increased soft-tissue density surrounding the fracture site. This homogeneous, cloud-like appearance indicated activation of the early repair response. Further imaging analysis revealed that the fracture gap was filled with a soft cartilaginous callus composed of collagen fibers and cartilaginous matrix synthesized by proliferating and differentiating fibroblasts and chondrocytes. This soft callus presented as a typical soft-tissue-density structure without signs of mineralization, and its contour conformed to the anatomical shape of the fracture ends, achieving initial bridging and stabilization of the fracture. Additionally, no significant bone resorption or necrosis was observed in the main fracture fragments. The gap between the small free fragment and the main sesamoid body had narrowed slightly compared with the pre-treatment images, with no evidence of displacement or malunion. These findings indicated that the healing process was in the critical stage of soft-callus formation, consistent with the physiological characteristics of sesamoid bone fracture repair in horses.

#### 4.2.3. Post-Treatment Ultrasonographic Examination

**Figure 6:**
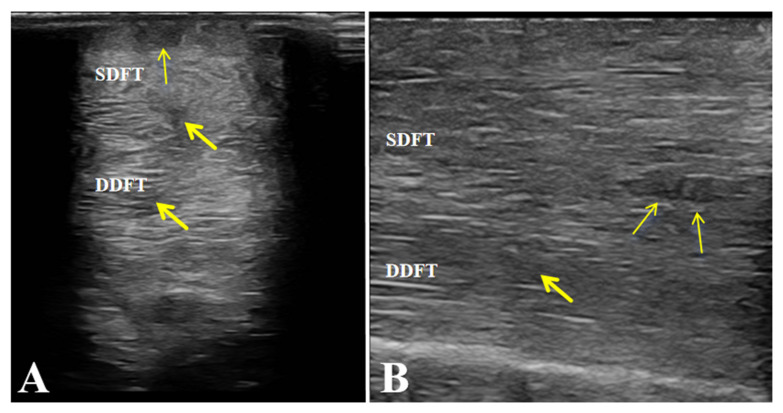
Post-treatment ultrasonographic images of the affected horse. (**A**,**B**) Increased echogenicity and uniform fibrillar alignment of the superficial digital flexor tendon (SDFT) and deep digital flexor tendon (DDFT), without fibrosis or adhesions. Arrows indicate the repaired areas of the tendon post-treatment.

After completing the medication combined with LIPUS-assisted therapy, ultrasonographic examination revealed marked improvement in the overall echogenicity of the affected SDFT and DDFT. Both tendons exhibited uniformly increased echogenicity, with no diffuse hypoechoic areas or regions of heterogeneous echotexture within the tendon substance. The internal homogeneity had become comparable to that of the contralateral healthy tendons. In the sagittal scans, the normal parallel, fibrillar echotexture was largely restored, showing well-organized, continuous fibers aligned with the longitudinal axis of the tendon, without evidence of disruption or fiber-pattern irregularity (Figure 6).

#### 4.2.4. Post-Treatment Hematological and Physiological Examinations

Hematological and physiological examinations were performed on the affected horse after drug and LIPUS therapy. The results showed that all hematological parameters returned to normal ranges, indicating resolution of inflammation and recovery of systemic function (Table 5).

#### 4.2.5. Post-Treatment Blood Biochemical Examinations

Blood biochemical examinations were conducted on the affected horse after drug and LIPUS therapy. The results showed that the levels of alkaline phosphatase (ALP), calcium (Ca), inorganic phosphorus (P), and calcium–phosphorus product (Ca × P) were all increased, indicating enhanced osteoblast activity, which promoted calcium salt deposition and was beneficial for fracture healing (Table 6).

#### 4.2.6. Post-Treatment Inflammatory Cytokine Detection

Serum inflammatory cytokine assays were performed on the affected horse after drug and LIPUS therapy. The results showed that the levels of tumor necrosis factor-α (TNF-α), interleukin-1β (IL-1β), and interleukin-6 (IL-6) returned to normal ranges, indicating that the inflammatory response caused by the fractures and tendon injuries was resolved, which accelerated fracture healing (Table 7).

Overall, after combined treatment with NSAIDs, antibiotics, intra-articular therapy, and LIPUS, the affected horse showed radiographic and ultrasonographic evidence of healing, as well as normalization of hematological and biochemical parameters, with no recurrence of flexor tendinitis. Radiographs demonstrated progressive healing of the proximal sesamoid bone fracture, confirming the therapeutic effectiveness of LIPUS-assisted therapy.

## 5. Discussion

Treatment options for minor fractures in racehorses include rest, external fixation, therapeutic cauterization, systemic administration of non-steroidal anti-inflammatory drugs (NSAIDs), resection of the suspensory ligament at the apex of the affected sesamoid bones, neurectomy, surgical removal of fracture fragments, and autologous cancellous bone grafting [24,25,26]. Typically, the proximal sesamoid bones of horses play a supportive role in the contraction of the flexor tendons behind the metacarpophalangeal joint. During intense exercise, the joint flexes excessively, and the sesamoid bones are subjected to compressive forces between the third metacarpal bone, proximal phalanx, and adjacent soft tissues. Consequently, pressure can increase on the suspensory ligaments, particularly at the apex and lateral margins of the sesamoid bones, which may impair local blood circulation, reduce bone mineral density, and thereby increase the risk of PSBF and secondary flexor tendinitis [27,28,29]. Forelimb flexor tendinitis has been reported to account for 6–13% of all musculoskeletal injuries in racehorses, with most studies linking its occurrence to racing or other high-intensity athletic activities [30,31]. The Yili horse in this study developed lameness and swelling of flexor tendons due to high-intensity competition and was subsequently diagnosed with flexor tendinitis secondary to PSBF based on clinical and imaging examinations.

Preliminary diagnosis of flexor tendinitis secondary to PSBF can usually be achieved through medical history review, visual inspection during rest and movement, palpation, and local nerve block tests, among which, local nerve block helps accurately identify the lesion [32]. In this case, radiography and ultrasonography played a key role in confirming the diagnosis, supported by hematological and biochemical analyses. The imaging findings showed a fracture at the apex of the proximal sesamoid bone, accompanied by inflammation of the SDFT and DDFT. Blood tests revealed elevated NEU, WBC, and LYM, indicating a systemic inflammatory response secondary to fracture and tendon injury, whereas RBC and HGB levels were decreased, suggesting bone marrow suppression.

After a fracture, osteoblast activity increases, leading to the secretion of ALP, which elevates the concentration of local inorganic phosphorus, thereby promoting calcium salt deposition and hydroxyapatite remodeling—key process in fracture healing [33]. Calcium (Ca) and phosphorus (P) in the serum interact with each other, and their product (Ca × P) is maintained within a certain range. When Ca × P exceeds the saturation point of Ca_3_(PO_4_)_2_ and CaCO_3_, calcium phosphate begins to nucleate and form a precipitate which subsequently becomes mineralized as Ca_3_(PO_4_)_2_ and CaCO_3_, which are deposited in the bone as inorganic components of bone [34]. Therefore, ALP, Ca, P, and Ca × P can be used as reliable biochemical markers to evaluate osteoblast activity and fracture healing progression. Similarly, CK and LDH are recognized as markers of muscle injury, reflecting the extent of tissue damage and repair [35].

In this case, treatment with NSAIDs combined with adjuvant LIPUS therapy led to notable increases in ALP, Ca, P, and Ca × P levels compared with their pre-treatment values, indicating enhanced osteoblast metabolism and accelerated fracture healing. Meanwhile, serum CK and LDH levels decreased significantly compared with pre-treatment, implying reduced muscle activity based on lameness. Together, these findings suggest that LIPUS, when combined with NSAIDs, can stimulate bone metabolism, improve tissue regeneration, and mitigate inflammatory responses.

Proinflammatory cytokines, such as IL-1β, IL-6, and TNF-α, play central roles in fracture-associated inflammation and bone metabolism regulation [36]. Specifically, TNF-α is a pro-inflammatory cytokine that binds to tumor necrosis factor receptor-1 (TNFR-1), thereby promoting the production of other cytokines. IL-1β, primarily produced by macrophages and endothelial cells, stimulates the activation of T lymphocytes and secretion of B-cell antibodies, thereby amplifying inflammation. IL-6, as a pro-inflammatory cytokine, recognizes and binds to IL-6 receptors on the surface of target cells to form a signal-transducing complex that regulates cytokine expression [37,38].

In this study, the serum levels of TNF-α, IL-1β, and IL-6 of the affected horse were raised before treatment, indicating that these cytokines are involved in the early inflammatory response associated with PSBF and tendon injury. After treatment, their levels returned to referenced ranges, suggesting that NSAIDs combined with LIPUS effectively reduced inflammatory cytokine expression, alleviated fracture-related inflammation, and accelerated and tendon healing.

Currently, clinical management of PSBF-related flexor tendinitis varies widely and includes the use of osteoclast inhibitors, intra-articular injection into the MTPJ, orthopedic plate fixation, and radial pressure wave therapy [39]. In instances with small free bone fragments, surgical excision may be considered, and arthroscopic minimally invasive procedures have been adopted in some international equine clinics. However, such invasive techniques may cause secondary injury to the suspensory ligaments and tendon structures surrounding the sesamoid bone [20]. To avoid the risks of secondary trauma and infection associated with surgery, this study employed a non-invasive strategy consisting of NSAID blockade therapy combined with LIPUS intervention, offering both safety and effectiveness.

Previous studies have confirmed the central role of LIPUS in promoting fracture repair. According to Rawool et al. [40], LIPUS significantly increases vascular perfusion at the fracture region, an essential determinant of the healing process. Older horses, due to vascular sclerosis and reduced metabolic activity, typically have markedly poorer perfusion than younger horses. Clinical observations suggest that LIPUS combined with NSAIDs may help improve blood supply at lesion sites, thereby providing enhanced nutritional support for bone healing, and may offer potential adjunctive benefits in alleviating the delayed healing commonly observed in aged horses. Moreover, LIPUS enhances integrin expression, facilitates osteoblast adhesion and proliferation at the fracture site, and modulates bone-repair-related gene expression, thereby accelerating the healing process [41]. As a continuously advancing biophysical therapy, LIPUS optimizes bone healing while minimizing thermal effects, achieving precise regulation of bone formation through molecular, cellular, and biomechanical interactions [42]. In the field of tendon repair, LIPUS has demonstrated clear advantages as well, including improvement of the early healing microenvironment of flexor tendon tears, reduction in inflammatory cell infiltration, and enhancement of functional range of movement at the repair site [43,44]. In clinical practice, LIPUS is often incorporated as part of a combination therapy in tendon repair interventions, with its therapeutic effects being closely dependent on the synergy of the overall treatment regimen.

It is important to note that single therapy has inherent limitations in the management of PSBF-associated flexor tendinitis. NSAIDs effectively alleviate inflammatory pain symptoms, yet they lack direct regenerative effects on bone and tendon tissue; long-term use may also increase the risk of chronic injury, resulting in a “treating the symptoms but not the cause” dilemma [45]. Conversely, LIPUS can effectively promote tissue regeneration but is insufficient for rapid suppression of acute inflammatory cascades, creating a “treating the cause but with delayed effect” limitation [46]. The combined therapy proposed in this study offers an innovative solution to these clinical pain points. First, to reconcile the adverse-effect risks of prolonged NSAID use and the slower onset of LIPUS, we adopted an optimized design of “short-term, moderate-dose NSAIDs + full-course LIPUS repair.” This allows the duration of NSAID administration to be reduced while maintaining adequate anti-inflammatory efficacy, thereby significantly lowering the risk of gastrointestinal or renal complications. Moreover, NSAID-mediated inflammation control may act synergistically with low-intensity pulsed ultrasound to promote the initiation of tissue repair, helping to initiate the healing process at an earlier stage [47,48]. Second, to address the high risk of chronic flexor tendinitis associated with fractures at the base of the metacarpal bones, the combined therapy simultaneously promotes cartilaginous callus formation and the orderly remodeling of tendon collagen, thereby enhancing fracture healing outcomes, reducing tendon adhesions, and overcoming the traditional therapeutic limitation in which symptoms are readily alleviated but functional recovery remains difficult [49,50,51]. Third, the non-invasive nature of LIPUS combined with oral NSAIDs provides a dual bone-tendon repair strategy without requiring surgical intervention, offering an attractive alternative for mild cases unsuitable for surgery or for postoperative rehabilitation, thereby expanding treatment options for musculoskeletal injuries [52,53].

In this study, a single Yili horse diagnosed with PSBF-induced flexor tendinitis triggered by exercise overload underwent comprehensive evaluation, including lameness scoring, imaging examinations, and hematological testing. Based on the confirmed etiology and specific lesion location, a targeted treatment plan was made. After receiving combined NSAID and LIPUS therapy, the horse showed improved local blood supply and enhanced fracture healing, and it ultimately achieved a favorable clinical outcome. These observations suggest that LIPUS may have potential therapeutic utility in PSBF and associated flexor tendinitis, and that its combined application with NSAIDs may offer both efficacy and safety. As an initial attempt at integrating NSAIDs with LIPUS for this condition, this study provides a new exploratory direction, though its potential clinical value requires further validation in a larger sample size. In future studies, larger sample sizes and rigorously designed randomized controlled trials should be employed, with explicit exclusion of confounding interventions, appropriate control groups, extended follow-up periods, and standardized evaluation criteria and methodologies. These approaches will allow more scientific and objective validation of the independent efficacy of LIPUS, as well as the effectiveness and safety of combined NSAID–LIPUS therapy, thereby providing reliable evidence-based support for the clinical management of this condition.

## Figures and Tables

**Table 1:** Lameness grades and scores of horses given exercise.

Grade	Clinical Manifestation	Score
**I.** Normal	No signs of lameness; gait appears completely normal.	0
**II.** Minor	Mild or intermittent lameness, usually not apparent unless under specific conditions such as under saddle or on a circular track with a hard surface.	1
**III.** Moderate	Lameness not apparent at a walk or in straight-line trotting, but consistently present under certain conditions (e.g., lunging).	2
**IV.** Claudication	Lameness clearly visible during straight-line trotting and persistent.	3
**V.** Severe	Lameness observable even at a walk.	4
**VI.** Grievous	The horse is non-weight-bearing on the affected limb and unwilling or unable to walk.	5

**Table 2:** Routine blood test results of the affected horse.

Parameter	Results	Reference Range
WBC (×10^9^/L)	12.98↑	5.0–12.0
NEU (×10^9^/L)	7.23↑	2.18–6.96
LYM (×10^9^/L)	6.01↑	1.32–5.86
EOS (×10^9^/L)	0.59	0.01–1.00
BASO (×10^9^/L)	0.011	0–0.12
MONO (×10^9^/L)	0.57	0.05–0.92
RBC (×10^12^/L)	5.05↓	5.3–10.5
HGB (mmol/L)	97.77↓	100–170
HCT (L/L)	29.66↓	30–49
MPV (fL)	5.46↓	5.5–8.0

Note: Arrows visually denote increasing and decreasing trends in the disease index.

**Table 3:** Blood biochemical test results of the affected horse.

Parameter	Results	Reference Range
ALP (U/L)	269.12	50–300
CK (U/L)	328.45↑	50–300
LDH (U/L)	553.46↑	100–500
Ca (mmol/L)	2.68	2.2–3.0
P (mmol/L)	1.39	0.7–1.5
Ca × P (mmol/L)	3.73	2.5–5.0

Note: Arrows visually denote increasing and decreasing trends in the disease index.

**Table 4:** Serum ELISA test results of the affected horse.

Parameter	Results (pg/mL)	Reference Range
TNF-α	260.3↑	0–50
IL-1β	302.5↑	5–50
IL-6	206.1↑	10–100

Note: Arrows visually denote increasing and decreasing trends in the disease index.

**Table 5:** Hematological and physiological examination results of the affected horse after treatment.

Parameter	Results	Reference Range
WBC (×10^9^/L)	11.25	5.0–12.0
NEU (×10^9^/L)	4.36	2.18–6.96
LYM (×10^9^/L)	5.27	1.32–5.86
EOS (×10^9^/L)	0.56	0.01–1.00
BASO (×10^9^/L)	0.1	0–0.12
MONO (×10^9^/L)	0.54	0.05–0.92
RBC (×10^12^/L)	5.52	5.3–10.5
HGB (mmol/L)	122.92	100–170
HCT (L/L)	44.15	30–49
MPV (fL)	5.87	5.5–8.0

**Table 6:** Blood biochemical examination results of the affected horse after treatment.

Parameter	Results	Reference Range
ALP (U/L)	335.7↑	50–300
CK (U/L)	261.47	50–300
LDH (U/L)	411.49	100–500
Ca (mmol/L)	3.7↑	2.2–3.0
P (mmol/L)	1.61↑	0.7–1.5
Ca × P/(mmol/L)	5.96↑	2.5–5.0

Note: Arrows visually denote increasing and decreasing trends in the disease index.

**Table 7:** Serum inflammatory cytokine levels of the affected horse after treatment.

Parameter	Results (pg/mL)	Reference Range
TNF-α	39.3	0–50
IL-1β	41.7	5–50
IL-6	84	10–100

## Data Availability

The original contributions presented in this study are included in the article. Further inquiries can be directed to the corresponding authors.
